# Mental Health and Contraceptive Knowledge in High Schoolers: Comparing Remote and In-Person Learning during COVID-19

**DOI:** 10.3390/medicina59101876

**Published:** 2023-10-22

**Authors:** Denisa Hinoveanu, Doru Mihai Anastasiu, Cosmin Citu, Doru Ciprian Crisan, Zoran Laurentiu Popa, Nicoleta Nicolae, Catalin Dumitru, Oana Neda-Stepan, Roxana Manuela Fericean, Lavinia Stelea

**Affiliations:** 1 Department of Obstetrics and Gynecology, “Victor Babes” University of Medicine and Pharmacy Timisoara, 300041 Timisoara, Romania; adriana.hinoveanu@umft.ro (D.H.); doru_anastasiu@yahoo.com (D.M.A.); citu.ioan@umft.ro (C.C.); crisan.doru@umft.ro (D.C.C.); nicolae.nicoleta@umft.ro (N.N.); dumitru.catalin@umft.ro (C.D.); stelea.lavinia@umft.ro (L.S.); 2 Doctoral School, “Victor Babes” University of Medicine and Pharmacy, Eftimie Murgu Square 2, 300041 Timisoara, Romania; oanastepan@yahoo.com (O.N.-S.); manuela.fericean@umft.ro (R.M.F.); 3 Department VIII—Neurosciences, Discipline of Psychiatry, “Victor Babes” University of Medicine and Pharmacy, Eftimie Murgu Square 2, 300041 Timisoara, Romania; 4 Department of Infectious Diseases, “Victor Babes” University of Medicine and Pharmacy Timisoara, 300041 Timisoara, Romania

**Keywords:** contraception, online education, depression, anxiety, teenagers

## Abstract

*Background and Objectives*: In response to the COVID-19 pandemic’s effects on education, this study delves into the behavioral, mental health, and sexual education characteristics of high school students during 2020–2021 and 2022–2023. *Materials and Methods*: We evaluated a variety of factors, including substance use, academic performance, sexual activities, mental health support, pandemic-related anxiety levels, and quality of life indicators using standardized instruments such as the SF-36, GAD-7, and WHOQOL-BREF. Furthermore, we addressed specific questions concerning contraception and sexual education during this period. *Results*: The questionnaires were filled in by 44 students in 2020–2021 and 41 students in 2022–2023. Significant findings included a noteworthy increase in COVID-19 vaccination rates, from 18.2% in 2020–2021 to 39.0% in 2022–2023 (*p* = 0.033), enhanced perceptions of mental health support during remote learning, from 7.1% to 20.0% (*p* = 0.044), and a rise in students partaking in reproductive health discussions from 10.7% to 25.0% (*p* = 0.046). Additionally, there was a marked decline in anxiety regarding the transition back to in-person learning (*p* = 0.048). Health surveys, such as the SF-36, signaled improvements in both physical and mental health over the years (*p* = 0.046 and *p* = 0.019, respectively), while the GAD-7 scores depicted a considerable reduction in anxiety symptoms (*p* = 0.038). The WHOQOL-BREF results also highlighted a significant uptick in students’ mental well-being in 2022–2023 (*p* = 0.039). *Conclusions*: As the COVID-19 pandemic ended, high school students exhibited shifts in behavior, health, and education over four academic years, particularly in areas of contraceptive knowledge and mental health outcomes. The pronounced enhancements in vaccination rates, perceptions of mental health support, participation in health conversations, and overall mental wellness emphasize the adaptability and resilience of students in these tumultuous periods, and a general increase in contraceptive knowledge and quality of life during the end of the pandemic.

## 1. Introduction

The COVID-19 pandemic, an unprecedented global health crisis, reshaped various dimensions of society, from healthcare systems to educational infrastructures [1,2,3,4]. A prominent transition instigated by this crisis was the abrupt shift to remote learning, attempting to curb the spread of the virus while maintaining educational processes. However, this digital shift had multifaceted implications that reached beyond the academic sphere [5,6].

High school students, being at a transitional age, are particularly vulnerable to the nuances of these changes, on top of the transformations their body suffers during the teenage period [7,8]. Their sexual and reproductive health, a pivotal aspect of this life stage, is deeply influenced by their knowledge and understanding of contraception and sexual education [9,10]. Within Romania, the need for robust contraceptive education is amplified considering the country’s above-average teenage pregnancy rates within the European context [11,12]. Coupled with limited or no sexual education in schools during the COVID-19 pandemic, and challenges in accessing contraceptives, there exists a critical gap that demands attention [13]. Nevertheless, the pandemic imposed important strains on educational systems and healthcare accessibility that might have impacted the contraceptive knowledge among high school students [14,15].

Beyond the domain of reproductive health, the pandemic’s mental toll on the global population, especially adolescents, has been undeniable [16,17]. Factors such as isolation, uncertainties about the future, and the challenge of adapting to new modes of learning have potential implications on anxiety and depression levels [18,19,20]. For high school students in Romania, the intersection of these mental health challenges with their reproductive health knowledge, especially during the back-and-forth transition of learning modes, remains an under-researched domain [21].

Given these concerns, the current study aims to find any potential changes in contraceptive knowledge among high school students as they navigated through different learning environments, by comparing the online education methods with the regular in-person education methods. Moreover, it seeks to understand the mental well-being of these students during these changing times, employing standardized and unstandardized questionnaires, such as the SF-36, GAD-7, and the WHOQOL-BREF, to assess their anxiety and depression levels.

## 2. Materials and Methods

### 2.1. Research Design and Ethical Considerations

The study employed a cross-sectional research design to analyze the contraceptive knowledge and mental health outcomes of high school students during the transitional phase of remote to in-person learning between the years 2020 and 2023. Collaborative efforts were made with numerous high schools across Romania to facilitate the research process. In line with rigorous ethical standards, the project received approval from the Local Commission of Ethics for Scientific Research, which is in strict accordance with the EU GCP Directives 2005/28/EC, ICH guidelines, and the principles detailed in the Declaration of Helsinki.

### 2.2. Inclusion Criteria

The process of selecting participants started by contacting high school principals during the pandemic period 2020 to 2023, in order to determine potential issues and students willing to answer the surveys. Potential participants were identified based on demographic variables. Eligible students were those aged between 16 and 19 who willingly participated in the study, as evidenced by their signed informed consent forms. For each academic year being studied, approximately 100 students were chosen, ensuring a substantial sample size for a robust comparative analysis across the transitional years. Exclusion criteria encompassed students who refused participation, those who did not agree to consent, and those who did not correctly fill in the questionnaires.

### 2.3. Variables

The decision to compare high school students between 2020–2021 with others from 2022–2023 period was influenced by the switch from the online school system during the first two years of the pandemic, to the traditional in-person teaching system. The study comprehensively assessed various attributes, including the age of participants, grade level, socioeconomic background, substance use habits, and prior exposure to sexual education. A primary focus was directed towards their knowledge, attitudes, and practices concerning contraception. Alongside this, the research analyzed students’ mental health states, using their responses to the WHOQOL-BREF, GAD-7, and SF-36 questionnaires. Moreover, a tailored survey was designed to solicit responses to pandemic-specific queries. The objective was to recognize the interplay between contraceptive understanding and mental health shifts during the observed period. All collected data were online and were anonymized according to EU GDPR requirements. All other students’ background data were collected using the same online forms.

### 2.4. Surveys Employed

The investigation incorporated several established instruments to dissect the multifaceted experiences of the surveyed students. The Short Form-36 (SF-36) [22,23] was utilized to assess health-related quality of life (HRQOL) and functional status across essential domains like physical, social, and mental well-being. Scores ranged from 0 to 100, reflecting the quality of life, while higher scores indicated better health status and quality of life. The WHOQOL-BREF, consisting of 26 items, provided another lens to gauge the quality of life [24,25]. This tool encompasses four dimensions of quality of life, including physical health, mental health, a social domain, and an environmental domain. Higher WHOQOL scores indicate better quality of life. To delve deeper into the mental health realm, the Generalized Anxiety Disorder-7 (GAD-7) [26,27] tool was incorporated to measure symptoms of generalized anxiety and depression severities, respectively. Higher GAD-7 scores indicate higher anxiety symptoms. In addition to these standardized tools, a unique 12-question survey was crafted to identify specific differences related to the COVID-19 pandemic and its impact on the students’ education and knowledge, without being used as a psychometric tool.

### 2.5. Statistical Analysis

Data management and analysis were conducted utilizing the statistical software SPSS version 26.0 (SPSS Inc., Chicago, IL, USA). The sample size was calculated based on a convenience sampling method, with a minimum of 80 respondents at a 95% confidence level and 10% margin of error. Continuous variables were represented as mean ± standard deviation (SD), while categorical variables were expressed in terms of frequencies and percentages. To analyze the changes between two means of continuous variables, Student’s *t*-test was utilized. The Chi-square test was utilized for the categorical variables. A *p*-value threshold of less than 0.05 was set for statistical significance. All results were double-checked to ensure accuracy and reliability.

## 3. Results

In assessing the background characteristics of high school students during the specified years, distinct patterns were observed. A comparison of the age of participants from the two academic years, collecting data from 44 students in 2020–2021 and 41 students in 2022–2023, showed a marginal difference. The mean age during 2020–2021 was 17.5 years with a standard deviation of 1.3 years, whereas for 2022–2023, it was slightly higher at 17.8 years with a standard deviation of 1.2 years; however, this difference was not statistically significant (*p* = 0.273). Breaking down age further, in both academic years, the proportion of students in the age range of 16–17 and 18–19 years was comparable, with 56.8% versus 56.1% and 43.2% versus 43.9%, respectively, and these differences were not significant (*p* = 0.946).

When evaluating substance use behavior, smoking was slightly more prevalent in 2020–2021, with 20.5% of students reporting it, compared to 12.2% in 2022–2023, but the difference was not statistically significant (*p* = 0.304). Alcohol consumption was reported by 27.3% of students in 2020–2021 and 24.4% in 2022–2023 (*p* = 0.761). The use of drugs was reported by a smaller percentage of students, 6.8% in 2020–2021 and 9.8% in 2022–2023, a difference that was not statistically significant (*p* = 0.622).

Regarding the place of origin, a higher proportion of students in both years originated from urban areas, with 84.1% in 2020–2021 and 78.0% in 2022–2023. The slight decline in urban-origin students in 2022–2023 was not statistically significant (*p* = 0.476). Considering high school grade, the distribution between the 10th, 11th, and 12th grades was nearly even for both years, with no significant difference (*p* = 0.839). Notably, there was a marked increase in the number of students who had received the COVID-19 vaccination from 2020–2021 (18.2%) to 2022–2023 (39.0%). This increase was statistically significant (*p* = 0.033). Lastly, regarding commencement of sexual activity, there was a slight increase in the percentage from 50.0% in 2020–2021 to 58.5% in 2022–2023, though this increase was not statistically significant (*p* = 0.429), as seen in Table 1.

During remote learning, only a minor fraction of students in 2020–2021 (12.5%) and 2022–2023 (8.3%) reported receiving primary contraception information from online classes. This difference was not statistically significant (*p* = 0.461). Challenges accessing contraceptive information during remote learning were noted by 21.4% of the participants in 2020–2021 and a slightly higher number, 26.7%, in 2022–2023; however, this increase was also not statistically significant (*p* = 0.510).

Regarding mental health support, 7.1% of students from 2020–2021 believed that the support provided during remote learning was as adequate as that during in-person school sessions. This perception significantly increased to 20.0% in 2022–2023 (*p* = 0.044). Additionally, there was a notable difference in engagement in discussions about reproductive health during remote learning. A significant rise from 10.7% in 2020–2021 to 25.0% in 2022–2023 reported greater engagement during remote sessions compared to in-person classes (*p* = 0.046).

When questioned about the impact of the pandemic’s uncertainty and stress on contraceptive decision-making, the average score in 2020–2021 was 7.3 (SD = 3.9) on a scale of 1 to 10, which decreased marginally to 6.0 (SD = 3.4) in 2022–2023, but this decline was not statistically significant (*p* = 0.106). Anxiety about returning to in-person learning was higher in 2020–2021 with an average score of 7.5 (SD = 4.4) than in 2022–2023, which averaged at 5.8 (SD = 3.3), and this reduction was statistically significant (*p* = 0.048).

In terms of resources provided, 30.4% in 2020–2021 reported being offered specific resources like websites, apps, or school programs, a percentage that dropped to 21.7% in 2022–2023, though the decline was not significant (*p* = 0.285). Students’ perception of how well their school addressed contraception education during the pandemic averaged 4.1 (SD = 3.5) in 2020–2021 and slightly improved to 4.9 (SD = 4.0) in 2022–2023; however, this difference was not statistically significant (*p* = 0.328).

A notable 55.4% of the participants from 2020–2021 believed that their mental health status during the pandemic influenced their understanding of contraceptive education. This decreased to 40.0% in 2022–2023, though the decrease was not statistically significant (*p* = 0.098). Additionally, 19.6% in 2020–2021 and 26.7% in 2022–2023 observed changes in peers’ attitudes or behaviors concerning contraception and reproductive health discussions, but this difference was not significant (*p* = 0.371).

Students in 2020–2021 rated the effect of the mode of learning on their understanding of contraception at an average of 6.7 (SD = 4.1) on a scale of 1 to 10, while those in 2022–2023 rated it slightly lower at 6.0 (SD = 3.5), a difference which was not statistically significant (*p* = 0.390). Lastly, the importance of consistent contraceptive education provision, irrespective of the learning mode, was rated at 6.9 (SD = 5.0) in 2020–2021 and was higher at 8.3 (SD = 3.6) in 2022–2023, but this increase was not significant (*p* = 0.145), as presented in Table 2.

An analysis of the SF-36 and GAD-7 survey results illustrated the variations in health status, quality of life, and anxiety symptoms between high school students in the academic years 2020–2021 and 2022–2023. The SF-36 survey, which assesses health status and quality of life, demonstrated a significant improvement in the scores from 2020–2021 to 2022–2023. For the physical component of the SF-36, the mean score in the academic year 2020–2021 was 52.9 (SD = 7.2) and this increased to 56.0 (SD = 6.9) in 2022–2023 (*p* = 0.046). This suggests a notable betterment in the physical health status of students in the latter academic year. In terms of the mental component, the mean score in 2020–2021 was 51.6 (SD = 6.8) and this witnessed an upliftment to 55.2 (SD = 7.1) in 2022–2023, indicating a statistically significant enhancement in mental health status (*p* = 0.019). Considering the total SF-36 scores, students in 2020–2021 had a mean of 53.0 (SD = 7.6), which rose to 56.4 (SD = 7.7) in 2022–2023, thus marking a statistically significant improvement in overall health and quality of life (*p* = 0.043).

**Figure 1:**
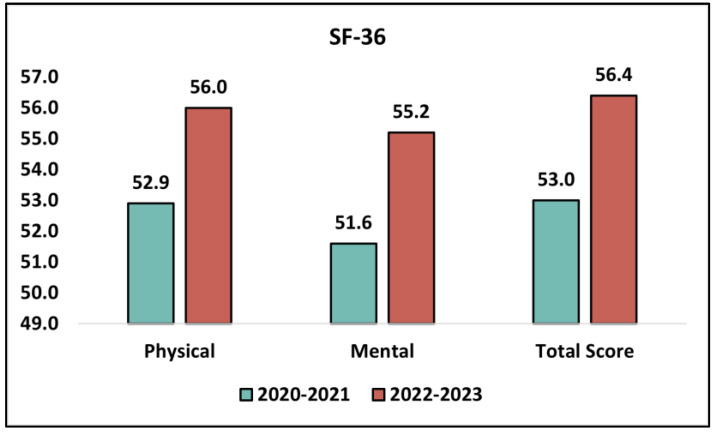
Analysis of the SF-36 questionnaire results.

Furthermore, the GAD-7 scores, which measure anxiety symptoms, demonstrated a significant drop from 2020–2021 to 2022–2023. The mean score in 2020–2021 was 7.7 (SD = 3.9) and this decreased to 6.5 (SD = 3.0) in 2022–2023 (*p* = 0.038). This suggests a reduction in anxiety symptoms in students transitioning from remote to in-person learning in the latter academic year, as presented in Table 3 and Figure 1.

The WHOQOL-BREF survey, which evaluates the quality of life across different domains, was utilized to discern the perceived quality of life among high school students during the academic years 2020–2021 and 2022–2023. In the physical domain, there was an observed increase in the mean score, which stood at 60.8 (SD = 15.1) in 2020–2021 and increased to 66.5 (SD = 18.2) in 2022–2023. However, this improvement was not statistically significant with a *p*-value of 0.119. The mental domain revealed a statistically significant enhancement in students’ quality of life. In 2020–2021, the mean score was 59.4 (SD = 16.6), which escalated to 67.1 (SD = 17.3) in 2022–2023. This elevation was significant with a *p*-value of 0.039, indicating a notable positive shift in mental well-being among students in the more recent academic year.

**Figure 2:**
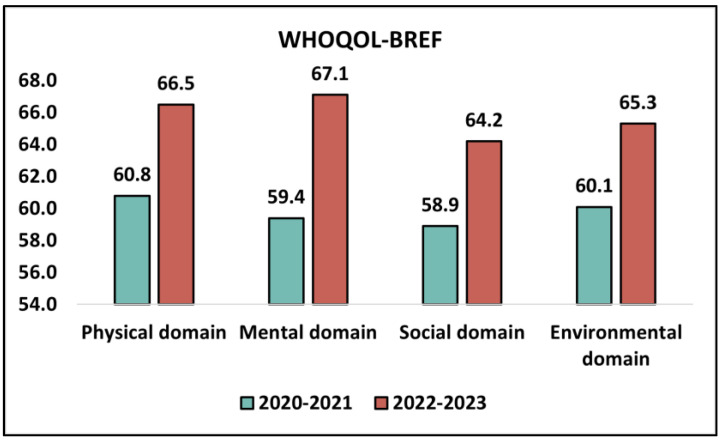
Average values of domain scores on the WHOQOL-BREF questionnaire.

In the social domain, students’ scores rose from a mean of 58.9 (SD = 17.5) in 2020–2021 to 64.2 (SD = 13.5) in 2022–2023. This increase, while indicating a possible improvement in social interactions and relationships, was not statistically significant as the *p*-value was 0.123. Lastly, the environmental domain, which encompasses factors like financial resources, freedom, safety, and health and social care accessibility, showed an upward trajectory in scores from 60.1 (SD = 13.8) in 2020–2021 to 65.3 (SD = 15.9) in 2022–2023. However, this rise was not statistically significant with a *p*-value of 0.110, as described in Table 4 and Figure 2.

## 4. Discussion

### 4.1. Important Findings and Literature Review

The impact of the COVID-19 pandemic on high school students’ mental health, contraceptive knowledge, and associated outcomes provides crucial insights into the challenges and changes experienced during the transition from remote to in-person learning. The background characteristics of students remained relatively consistent across both academic years, with slight fluctuations in substance use and a marked increase in COVID-19 vaccination rates. The substantial increase in vaccination from 2020–2021 to 2022–2023 underscores the intensified health campaigns and vaccine accessibility during this period [28,29].

Substance use, a factor often correlated with mental health and socio-environmental influences, showed little significant variance between the two years of the pandemic, as described in other studies [30,31]. It is noteworthy, though, that the slightly higher prevalence of smoking in 2020–2021 could be attributed to coping mechanisms during the peak of the pandemic. While our study controlled for several variables, we cannot rule out the potential impact of larger societal attitudes, policy changes, or unmeasured confounders affecting substance use trends during the period. However, as the study data suggest, alcohol and drug use did not display considerable changes, pointing towards the possible stabilization of external stressors or the consistent nature of these behaviors among high school students. This observation may warrant further investigation into social policies and community support systems that could have played a role in these stabilizations.

One of the pressing concerns during remote learning was the provision of adequate contraceptive education [32]. The findings suggest that the majority of students did not receive primary contraceptive information from online classes in both years. This highlights a potential gap in the remote learning curriculum, stressing the need for more comprehensive reproductive health education in online platforms. However, external factors, such as changes in societal views on sexuality and contraception, might also influence these observations.

Concurrently, the challenges in accessing contraceptive information remained marginally consistent, again emphasizing the need for improved dissemination of such vital information. The ongoing pandemic may have redirected educational priorities, leaving reproductive health in the shadows. Given the profound implications of inadequate sexual education, this warrants policy interventions and the inclusion of more structured modules in remote learning platforms.

Mental health support during remote learning appeared to be a focal point of improvement, with students in 2022–2023 perceiving it as more adequate compared to their counterparts in the previous year. This observed improvement might be the result of specific school or community-based interventions, like increased availability of counseling services or peer support groups, which we recommend future studies delve deeper into. Moreover, the significant increase in engagement in discussions about reproductive health during remote learning in 2022–2023 might imply a more proactive approach to addressing reproductive health during this period, similar to what other research found at the end of the COVID-19 pandemic [33,34].

From a psychological standpoint, the decline in the anxiety about returning to in-person learning from 2020–2021 to 2022–2023 suggests that students became more accustomed to the “new normal” and possibly felt more prepared or less apprehensive about returning to physical classrooms, as recent studies suggested [35,36]. The variations in health status, quality of life, and anxiety symptoms further solidify this interpretation. However, it is essential to also consider how external factors, like the broader societal acceptance and adjustment to post-pandemic conditions, might have influenced these positive shifts in mental health metrics. Moreover, the significant improvement in the SF-36 scores from 2020–2021 to 2022–2023 denotes an enhancement in both physical and mental health. The notable decrease in the GAD-7 scores in 2022–2023 compared to 2020–2021 is also indicative of reduced anxiety symptoms [37].

The WHOQOL-BREF survey results corroborate the findings from the SF-36 and GAD-7 scores. Although the improvement in the physical, social, and environmental domains was not statistically significant, the meaningful increase in the mental domain’s score in 2022–2023 underlines the potential positive mental health outcomes during the transition phase. These combined findings underscore the multifaceted impact of the pandemic on high school students and accentuate the need for continued attention to their mental health, reproductive education, and overall well-being as the world transitions to post-pandemic normalcy.

Nevertheless, the concerning trends elucidated by the data collected during 2020–2023, which highlighted significant gaps in the dissemination of primary contraception information during remote learning, further underscore the imperative need for robust sexual education. Romania, in particular, battles with a notably high prevalence of teenage pregnancies and abortions within the European context [11]. A lack of consistent and comprehensive sexual education can lead to uninformed decisions by the youth, resulting in unintended pregnancies and associated health risks. The data from our study indicate that during these pandemic years, although there has been some improvement in mental health support and discussions about reproductive health, there remain crucial areas that need significant enhancement, primarily in the realms of contraceptive information and education.

Comparatively, an Italian study throws light on an emerging pattern across Europe where young individuals overwhelmingly resort to the web for sexual and reproductive health information [38]. This raises concerns about the accuracy and reliability of the information sourced, especially in the absence of standardized and regulated content. Policymakers need to recognize the shift towards digital sources and ensure the provision of accurate and evidence-based online resources for the youth.

Therefore, both the Romanian and Italian studies [39] emphasize the critical role of structured, timely, and comprehensive sexual education in shaping informed decisions among adolescents. While the patterns of seeking information differ, with Romanian students leaning more on remote learning and Italian ones on the web, the conclusion is clear: countries must prioritize the delivery of sexual and reproductive health education, integrating it into the curriculum irrespective of the learning mode, ensuring accessibility, relevance, and accuracy. Investing in sexual education not only aids in reducing teenage pregnancies and abortions but also instills a sense of responsibility, knowledge, and empowerment among the youth, enabling them to navigate their romantic and sexual lives with confidence and prudence.

### 4.2. Study Limitations

The present study, while robust in its comprehensive assessment of the shifts in contraceptive knowledge and mental well-being among high school students, is not without limitations. First and foremost, employing a cross-sectional design restricts our ability to infer causality between the observed variables and the outcomes, merely providing a snapshot in time. The methodology does not account for the potential influence of external factors or events that may have concurrently affected students’ mental health and contraceptive understanding. Furthermore, our reliance on self-reported measures raises concerns about potential response biases, with students either intentionally or unintentionally misrepresenting their experiences or perceptions. The participation criteria, although rigorous, might have excluded valuable insights from students outside the age bracket of 16 to 19 years. Additionally, the transition between online and in-person learning may present varying challenges and advantages for different individuals, making it difficult to generalize findings. Furthermore, since this study was primarily observational, establishing causality or ruling out potential confounders is challenging. Lastly, while the study involved collaboration with numerous high schools across Romania, the potential for regional or school-specific idiosyncrasies affecting the responses should be considered.

## 5. Conclusions

Throughout the academic years 2020–2021 and 2022–2023, high school students demonstrated consistent background characteristics, with a pronounced rise in COVID-19 vaccinations by 2022–2023. Substance use tendencies remained relatively stable with no significant statistical variance. By 2022–2023, a pivotal observation emerged with students reporting increased mental health support during remote learning and heightened engagement in reproductive health conversations. Health metrics showed noteworthy enhancements in both physical and mental well-being, complemented by a decline in anxiety levels during the latter academic year. The WHOQOL-BREF survey underlined substantial progress in the mental domain’s quality of life in this timeframe.

In light of these findings, it is evident that the transitional phase of the pandemic has had multifaceted implications on the mental and reproductive health education of high school students. The enhanced mental health support and increased engagement in reproductive health discussions signify a positive shift in the educational landscape, potentially fueled by the collective societal response to the challenges posed by the pandemic. These findings not only underscore the resilience and adaptability of high school students but also emphasize the urgent need for policies and educational strategies that prioritize mental health and reproductive education. In the broader societal context, amidst the persistent challenges of the COVID-19 pandemic, our study accentuates the profound importance of addressing the holistic well-being and comprehensive education of adolescents.

## Figures and Tables

**Table 1:** Background characteristics.

	2020–2021 (*n* = 44)	2022–2023 (*n* = 41)	*p*-Value *	95% CI	AOR (95% CI)
Age, years (mean ± SD) **	17.5 ± 1.3	17.8 ± 1.2	0.273	(16.9–18.1)	-
Age range			0.946		
16–17	25 (56.8%)	23 (56.1%)		(42.0%, 70.5%)	0.98 (0.52, 1.85)
18–19	19 (43.2%)	18 (43.9%)		(29.5%, 57.0%)	1.03 (0.52, 2.05)
Substance use behavior					
Smoking	9 (20.5%)	5 (12.2%)	0.304	(10.6%, 33.2%)	0.75 (0.40, 1.42)
Alcohol use	12 (27.3%)	10 (24.4%)	0.761	(18.9%, 38.1%)	0.88 (0.52, 1.48)
Drugs	3 (6.8%)	4 (9.8%)	0.622	(1.5%, 15.5%)	1.25 (0.58, 2.68)
Place of origin (urban)	37 (84.1%)	32 (78.0%)	0.476	(70.2%, 92.8%)	0.72 (0.31, 1.67)
High school grade			0.839		
10th grade	15 (34.1%)	14 (34.1%)		(21.0%, 49.2%)	1.00 (0.51, 1.98)
11th grade	13 (29.5%)	10 (24.4%)		(17.3%, 43.8%)	0.76 (0.37, 1.59)
12th grade	16 (36.4%)	17 (41.5%)		(23.2%, 51.5%)	1.25 (0.63, 2.47)
COVID-19 vaccinated	8 (18.2%)	16 (39.0%)	0.033	(10.2%, 48.7%)	1.45 (1.08, 3.40)
Commenced sexual activity	22 (50.0%)	24 (58.5%)	0.429	(36.7%, 63.3%)	1.41 (0.82, 2.44)

* Chi-square or Fisher’s exact test; ** Student’s *t*-test; SD—Standard Deviation; CI—Confidence Interval; and AOR—Adjusted Odds Ratio.

**Table 2:** Unstandardized survey results.

Questions	2020–2021 (*n* = 44)	2022–2023 (*n* = 41)	*p*-Value *	95% CI	AOR (95% CI)
During remote learning, did you primarily receive information about contraception from online classes? (Yes, %)	7 (12.5%)	5 (8.3%)	0.461	(5.7%, 22.4%)	1.25 (0.78, 2.05)
Did you face challenges accessing contraceptive information during remote learning? (Yes, %)	12 (21.4%)	16 (26.7%)	0.510	(15.6%, 29.7%)	1.35 (0.80, 2.27)
Was the mental health support provided during remote learning as adequate as during in-person school sessions? (Yes, %)	4 (7.1%)	12 (20.0%)	0.044	(2.3%, 15.5%)	1.95 (1.01, 3.76)
Compared to in-person classes, did you engage more in discussions about reproductive health during remote learning? (Yes, %)	6 (10.7%)	15 (25.0%)	0.046	(4.8%, 20.1%)	2.75 (1.12, 6.79)
On a scale from 1 (never) to 10 (frequently), how often did the uncertainty and stress from the COVID-19 pandemic influence your decision-making related to contraception?	7.3 ± 3.9	6.0 ± 3.4	0.106 **	(5.8, 8.8)	-
Rate your level of anxiety about returning to in-person learning, with 1 being “not anxious at all” and 10 being “extremely anxious”.	7.5 ± 4.4	5.8 ± 3.3	0.048 **	(6.2, 10.1)	-
Were there specific resources (websites, apps, school programs) that were offered to you? (Yes, %)	17 (30.4%)	13 (21.7%)	0.285	(20.2%, 42.1%)	0.78 (0.42, 1.47)
On a scale from 1 (very poorly) to 10 (very well), how well do you believe your school addressed contraception education during the pandemic?	4.1 ± 3.5	4.9 ± 4.0	0.328 **	(3.2, 5.0)	-
Do you believe that your mental health status during the pandemic influenced your perception and understanding of contraceptive education? (Yes, %)	31 (55.4%)	24 (40.0%)	0.098	(43.8%, 66.8%)	0.55 (0.30, 1.01)
Did you observe a change in the attitudes or behaviors of your peers concerning contraception and reproductive health discussions? (Yes, %)	11 (19.6%)	16 (26.7%)	0.371	(12.3%, 29.6%)	1.45 (0.72, 2.90)
On a scale from 1 (not at all) to 10 (extensively), how much do you believe the mode of learning (remote vs. in-person) affected your understanding of contraception?	6.7 ± 4.1	6.0 ± 3.5	0.390 **	(5.8, 7.6)	-
On a scale from 1 (not at all) to 10 (very much), how important is it for schools to ensure contraceptive education is consistently provided, irrespective of the mode of learning (remote or in-person)?	6.9 ± 5.0	8.3 ± 3.6	0.145 **	(5.5, 8.3)	-

* Chi-square or Fisher’s exact test; ** Student’s *t*-test.

**Table 3:** SF-36 and GAD-7 survey results.

Scores (Mean ± SD)	2020–2021 (*n* = 44)	2022–2023 (*n* = 41)	*p*-Value *
SF-36—Physical	52.9 ± 7.2	56.0 ± 6.9	0.046
SF-36—Mental	51.6 ± 6.8	55.2 ± 7.1	0.019
SF-36—Total	53.0 ± 7.6	56.4 ± 7.7	0.043
GAD-7	7.7 ± 3.9	6.5 ± 3.0	0.038

* Student’s *t*-test; SD—Standard Deviation; SF-36—Short Form Survey (higher scores indicate better health status and quality of life); and GAD—General Anxiety Disorder (higher scores indicate higher anxiety symptoms).

**Table 4:** WHOQOL-BREF survey results.

WHOQOL-BREF (Mean ± SD)	2020–2021 (*n* = 44)	2022–2023 (*n* = 41)	*p*-Value *
Physical domain	60.8 ± 15.1	66.5 ± 18.2	0.119
Mental domain	59.4 ± 16.6	67.1 ± 17.3	0.039
Social domain	58.9 ± 17.5	64.2 ± 13.5	0.123
Environmental domain	60.1 ± 13.8	65.3 ± 15.9	0.110

* Student’s *t*-test; SD—Standard Deviation; WHOQOL-BREF—Brief Version of the World Health Organization Quality of Life survey (higher scores indicate better quality of life).

## Data Availability

The data presented in this study are available on request from the corresponding author.
